# Co-pathology may impact outcomes of amyloid-targeting treatments: clinicopathological results from two patients treated with aducanumab

**DOI:** 10.1007/s00401-023-02631-8

**Published:** 2023-09-19

**Authors:** Lawren VandeVrede, Renaud La Joie, Sheena Horiki, Nidhi S. Mundada, Mary Koestler, Ji-Hye Hwang, Peter A. Ljubenkov, Julio C. Rojas, Gil D. Rabinovici, Adam L. Boxer, William W. Seeley

**Affiliations:** 1 Department of Neurology, Memory and Aging Center, University of California San Francisco, 675 Nelson Rising Lane, Suite 190, San Francisco, CA 94158 USA; 2 Department of Radiology and Biomedical Imaging, University of California San Francisco, San Francisco, CA USA

**Keywords:** Alzheimer’s disease, Amyloid, Lewy body disease, Aducanumab, Clinicopathological correlation

Aducanumab is a monoclonal antibody that targets amyloid-beta aggregates, with engagement demonstrated using amyloid-beta positron emission tomography (PET) [1, 7]. Aducanumab is one example of a class of amyloid-targeting antibodies proposed as treatment for early AD [4, 9], but few clinicopathological studies are available from patients given these treatments. One study reported clinicopathological evidence of plaque reduction and remnant plaque-associated microglia reactivity with a 4-month aducanumab-to-autopsy interval [5], and another study highlighted the increased risk of cerebral hemorrhage after thrombolytic therapy [6]. These autopsy studies emphasize the value of clinicopathological descriptions to understand treatment effects and guide clinical management. However, key unresolved questions include (1) neuropathological correlates of amyloid-lowering and (2) pathobiology underlying poor clinical response. Herein, we report the clinical course and autopsy findings from two patients treated with aducanumab who may shed light on the time course of amyloid lowering and the potential impact of co-pathology on clinical response.

Both patients were clinically evaluated at the University of California, San Francisco (UCSF) and enrolled in ENGAGE (NCT02477800). Patient 1 also enrolled in EMBARK (NCT04241068). Aducanumab administration, florbetapir (FBP) PET, and clinical assessments were performed as specified in the clinical trial protocol [1]. Additional data, including flortaucipir (FTP) PET, were obtained through the UCSF Alzheimer’s Disease Research Center (ADRC). PET and autopsy data were analyzed locally, detailed in Supplementary Information (SI).

Patient 1: A 77-year-old white man (*APOE-ε3/ε3*) with a 36 pack-year smoking history reported decreased processing speed and executive dysfunction starting at age 63 after delirium following cervical spine surgery. At 71, he developed progressive short-term memory loss and visuospatial dysfunction, confirmed on neuropsychological testing. MRI showed moderate temporoparietal and medial temporal atrophy (Fig. 1a). Amyloid PET was visually positive and FTP PET showed medial temporal lobe radiotracer uptake. He was diagnosed with mild cognitive impairment (MCI) due to AD.

**Fig. 1:**
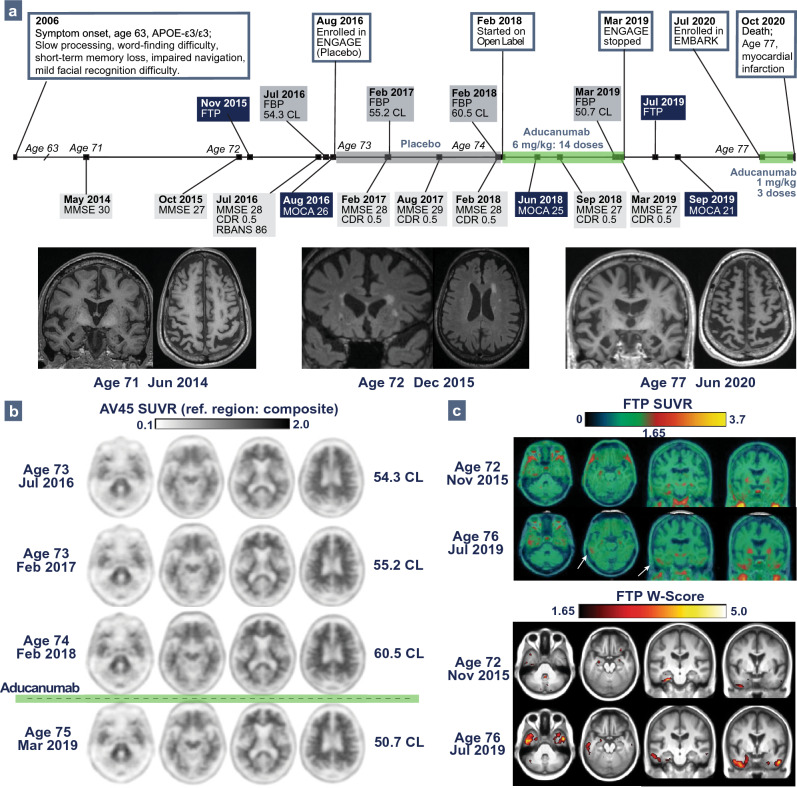
Patient 1’s Clinical Course and Neuroimaging. **a** Timeline of clinical course for Patient 1 with MRI demonstrating progression of hippocampal and biparietal atrophy, and white matter disease. **b** Florbetapir (FBP) PET throughout ENGAGE with centiloid values calculated using a composite reference region, including FBP after 12 months of treatment with low-dose aducanumab (fourth row). **c** Long-interval flortaucipir (FTP) PET showing transition from negative to positive (top rows, white arrows), and FTP PET referenced against cognitively normal control cohort highlight progression in temporal regions (bottom rows)

At 73, he enrolled in ENGAGE (randomized to placebo), and MMSE and CDR were unchanged during the trial. In the long-term extension, he received low-dose aducanumab (6 mg/kg), and one year later he remained clinically stable. FBP PET decreased to 16.2% (60.5 centiloids [CL] to 50.7 CL; Fig. 1b). He had no amyloid-related imaging abnormalities (ARIA). At 77, he enrolled in EMBARK re-dosing study. Clinically, he remained MCI stage with slight memory worsening. FTP PET medial temporal lobe signal increased, extending into inferolateral temporal regions (Fig. 1c) [2]. In EMBARK, he received three titration doses but died from cardiac arrest deemed unrelated to treatment. The cumulative aducanumab dose was 65 mg/kg.

Autopsy showed moderately developed AD (Fig. 2a–f, details in SI). The most severe degenerative changes involved temporal and limbic structures, and typical diffuse and neuritic amyloid plaques extended from the neocortex into the striatum. Amyloid plaque abundance and morphology were typical of changes at this clinicopathological stage, without reported changes to plaque morphology.^5^ Tau-positive neurofibrillary tangles were abundant in temporal and limbic regions, and sparse to moderate in neocortical areas associated with the transition from MCI to dementia. Additionally, chronic microinfarcts, with an associated fibrillary glial scar, were scattered throughout the subcortical white matter and deep gray structures.

**Fig. 2:**
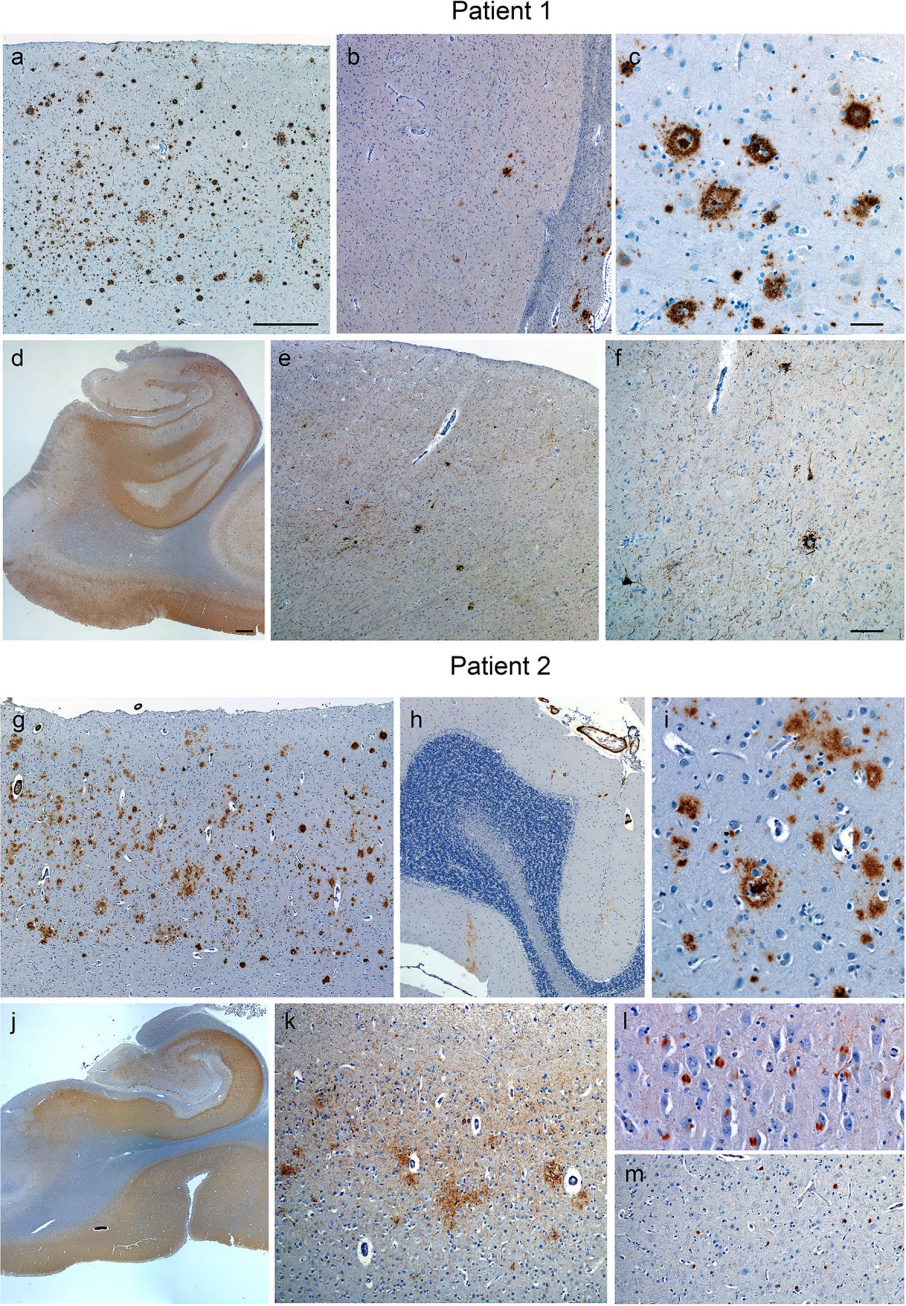
Neuropathological findings. Patient 1 showed diffuse and neuritic amyloid plaque deposition that reached from neocortical regions such as the middle frontal gyrus (**a**) to the striatum (**b**, putamen is shown). These plaques showed no evidence of partial clearance (**c**) as previously described [5]. Tau neurofibrillary pathology was substantial in mesial temporal (**d**), as well as basal temporal regions (not shown) and extended lightly into heteromodal association neocortices, such as middle frontal gyrus (**e** and **f**). Patient 2 showed more advanced ADNC, with amyloid plaques extending from middle frontal gyrus (**g**) to cerebellar molecular layer (**h**), without evidence of partial clearance (**i**), as well as moderate cerebral amyloid angiopathy (**g**, **h**). In parallel, Patient 2 showed more advanced tau neurofibrillary pathology, extending from medial temporal structures (**j**) to primary cortices, such a striate cortex (**k**). Additionally, Patient 2 showed diffuse neocortical LBD, affecting brainstem structures (not shown), limbic regions such as CA2 (**l**), and neocortical regions such as middle frontal gyrus (**m**). Scale bar in (**a**) represents 500 µM and applies to (**a**, **b**, **e**, and **g**). Scale bar in (**c**) represents 50 µM and applies to (**c**, **d**, and **l**). Scale bar in (**d**) represents 1000 µM and applies to (**d**, **h**, and **e**). Scale bar in (**f**) represents 100 µM and applies to (**f**, **k**, and **m**)

Patient 2: A 64-year-old white woman (*APOE-ε4/ε4*) developed cognitive decline at age 55, characterized by memory loss with repetitive questioning, word-finding trouble, and navigation difficulty, but also prominent anxiety and REM-behavior disorder (RBD; Fig. 3a) that suggested possible comorbid Lewy body disease (LBD). At 59, neuropsychological testing confirmed learning and memory impairment. MRI showed bilateral dorsal fronto-parietal atrophy and mild right hippocampal atrophy (Fig. 3b). Cerebrospinal fluid testing confirmed AD (ADmark^®^: Aβ_42_ 281.85 pg/mL, t-tau 555.4 pg/mL, p-tau181 88.2 pg/mL), and she was diagnosed with MCI due to early-onset AD.

**Fig. 3:**
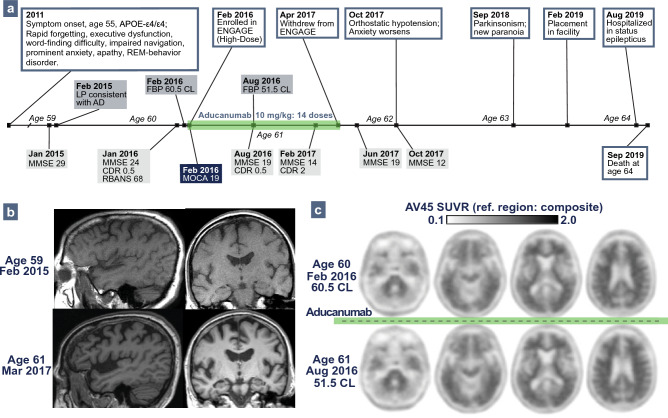
Patient 2 Clinical Course and Neuroimaging. **a** Timeline of clinical course for Patient 2. **b** Representative MRI slices demonstrating progression of atrophy. **c** Florbetapir (FBP) PET before (top) and after (bottom) receiving high-dose aducanumab through ENGAGE, with centiloid values calculated using a composite reference region

At 60, she enrolled in ENGAGE, randomized to high-dose aducanumab (10 mg/kg), and after six months FBP PET decreased to 14.9% (60.5 CL to 51.5 CL; Fig. 3c) [1, 7]. Nonetheless, her clinical symptoms worsened, especially anxiety. After one year (cumulative dose 100 mg/kg), she discontinued treatment due to clinical progression. She had no ARIA or treatment-related adverse events. Over the following years, she developed symptoms typical of dementia with Lewy bodies (DLB), including Parkinsonism, orthostatic hypotension, treatment-resistant anxiety, and paranoid delusions. At 64, she developed recurrent seizures and was hospitalized in status epilepticus, dying shortly thereafter.

Autopsy showed high ADNC (Fig. 2g–m**,** details in SI). Abundant amyloid-positive diffuse and neuritic plaques were seen throughout the cerebral cortex and extended caudally to the molecular layer of the cerebellum. No changes in plaque morphology were identified. Neurofibrillary tangles were abundant throughout limbic and cerebral cortical structures, including primary sensory, motor, and visual cortices. Importantly, diffuse neocortical Lewy body disease (LBD) was observed, extending to premotor and posterior parietal cortices. Finally, scarce and focal TDP-43 aggregation was found in the amygdala, and moderate amyloid angiopathy was seen in the cerebrum and cerebellum.

In summary, the cases reported here provide insight into the need for adequate dosing, while also suggesting that radiographic amyloid-lowering may be short-lived after drug discontinuation and less effective in the setting of comorbid neurodegenerative illnesses. In both cases, no evidence of amyloid plaque engagement was seen at autopsy despite modest amyloid-lowering on PET, possibly due to plaque re-accumulation given the long intervals between aducanumab discontinuation and autopsy. It remains uncertain whether amyloid removal can occur once, at an early clinical stage, or requires continued dosing to maintain clinical benefits.

For Patient 1, an impact on clinical course was difficult to discern, but cumulative dose was low and amyloid-lowering on PET was modest, remaining above the hypothesized 25 CL threshold sufficient for clinical effect. Interestingly, while Braak stage 5 tau is consistent with late-stage MCI, a Thal amyloid phase of 3 can be seen in individuals without substantial cognitive decline. However, prior studies correlating PET and Thal phase suggest 50–60 CL on amyloid PET is compatible with Thal phase 3–4 [3, 8], making it impossible to determine whether the postmortem Thal phase was lower than expected. Vascular co-pathology likely contributed to his clinical features, including the indolent course and frontal-subcortical dysfunction. The impact of vascular co-pathology on amyloid-targeting treatments, if any, is yet to be elucidated.

For Patient 2, well-developed AD and LBD were found at autopsy. Prominent comorbid LBD likely contributed to her RBD, Parkinsonism, anxiety, dysautonomia, and psychosis. The extensive tau neurofibrillary pathology suggests that either insufficient amyloid-lowering was achieved initially (remaining > 25 CL) or that tau spreading and propagation continue after amyloid-lowering unless maintenance dosing is administered. Additionally, *APOE-ε4* homozygotes may show less response to treatment [9]. Alternatively, LBD co-pathology may have contributed to her clinical progression despite amyloid reduction on PET. Further studies are needed to understand the impact of LBD co-pathology on amyloid-targeting treatments.

## Supplementary Information

Below is the link to the electronic supplementary material.

Supplementary file1 (DOCX 37 KB)

## Data Availability

The authors declare that the data supporting the findings of this study are available within the paper, its supplementary information files, and by request to the corresponding author.
