# Correction to: Co-pathology may impact outcomes of amyloid-targeting treatments: clinicopathological results from two patients treated with aducanumab

**DOI:** 10.1007/s00401-025-02855-w

**Published:** 2025-02-13

**Authors:** Lawren VandeVrede, Renaud La Joie, Sheena Horiki, Nidhi S. Mundada, Mary Koestler, Ji-Hye Hwang, Peter A. Ljubenkov, Julio C. Rojas, Gil D. Rabinovici, Adam L. Boxer, William W. Seeley

**Affiliations:** 1 Department of Neurology, Memory and Aging Center, University of California San Francisco, 675 Nelson Rising Lane, Suite 190, San Francisco, CA 94158 USA; 2 Department of Radiology and Biomedical Imaging, University of California San Francisco, San Francisco, CA USA

**Correction to: Acta Neuropathologica (2023) 146:777–781** 10.1007/s00401-023-02631-8

In this article, the cumulative dosage of aducanumab was incorrectly calculated due to failure to account for a change in the trial’s protocol. After review, the correct cumulative dose for Patient 1 is 67 mg/kg instead of 65 mg/kg, and the correct cumulative dose for Patient 2 is 68 mg/kg instead of 100 mg/kg and the patient was given aducanumab 6 mg/kg instead of 10 mg/kg.

