# Supplementary material for: Co-pathology may impact outcomes of amyloid-targeting treatments: clinicopathological results from two patients treated with aducanumab
Source: Acta Neuropathol. 2023 Sep 19;146(5):777–81. doi: 10.1007/s00401-023-02631-8 (PMC10564667; doi:10.1007/s00401-023-02631-8)
Supplement: Supplementary file 1 — Supplementary file1 (DOCX 37 KB) [file 401_2023_2631_MOESM1_ESM.docx]

**SUPPLEMENTARY INFORMATION**

**Supplemental Methods**

**Neuroimaging Methods**

MRI

MRI sequences were acquired at UCSF on either a 3T Siemens Tim Trio or a 3T Siemens Prisma Fit scanner: T1-weighted magnetization-prepared rapid gradient echo, fluid-attenuated inversion recovery (FLAIR), and susceptibility weighted imaging (SWI). Both scanners had very similar acquisition parameters (sagittal slice orientation; slice thickness, 1.0 mm; slices per slab, 160; in-plane resolution, 1.0 × 1.0 mm; matrix, 240 × 256; repetition time, 2300 ms; inversion time, 900 ms; flip angle, 9°), although echo time slightly differed (Trio, 2.98 ms; Prisma, 2.9 ms).

[18]F-Florbetapir

[18]F-Florbetapir PET were acquired at UCSF China Basin on a GE Discovery STE scanner. A low-dose CT scan was acquired for attenuation correction prior to PET acquisition, and PET data were reconstructed using an ordered subset expectation maximization algorithm with weighted attenuation and scatter correction. Radiotracer was injected intravenously as a bolus, and data analyzed in this paper were acquired for 20 min starting 50 min post injection. [18]F-Florbetapir-PET data was reconstructed as four 5-min frames.

T1 MRIs were segmented and parcellated using FreeSurfer version 7.1 (surfer.nmr.mgh.harvard.edu). PET frames were realigned using rigid-body transformation, averaged, and coregistered to their corresponding MRI using Statistical Parametric Mapping 12 (SPM12; Wellcome Department of Imaging Neuroscience, Institute of Neurology, London, UK). Standardized uptake value ratio (SUVR) images were created using a composite reference region that included the whole cerebellum, eroded white matter, and brainstem.[4] This composite reference region approach has shown greater reliability than other regions (e.g. cerebellum) for longitudinal analyses.[8] To obtain a measure of global cortical amyloid burden, we extracted a mean, cortical SUVR value for each tracer in native space using a weighted average of all FreeSurfer-derived cortical regions. [18]F-Florbetapir SUVR values were converted to Centiloid values using a previously validated equation.[1, 8]

[18]F-Flortaucipir

[18]F-Flortaucipir PET data were acquired at the Lawrence Berkeley National Laboratory (LBNL) on a single Siemens Biograph PET/CT scanner (Patient 1, 2015 scan) UCSF China Basin on a GE Discovery STE scanner (Patient 1, 2019 scan). A low-dose CT scan was acquired for attenuation correction prior to PET acquisition, and PET data were reconstructed using an ordered subset expectation maximization algorithm with weighted attenuation and scatter correction [18]F-Flortaucipir radiotracer was injected intravenously as a bolus, and data analyzed in this paper were acquired for 20 min starting 80 min post injection. [18]F-Flortaucipir-PET data was reconstructed as four 5-min frames.

The MRI closest in time to each PET time point was segmented and parcellated using FreeSurfer version 5.3 (https://surfer.nmr.mgh.harvard.edu/). PET frames were realigned using rigid-body transformations, averaged, and coregistered to their corresponding MRI using Statistical Parametric Mapping 12 (SPM12; Wellcome Department of Imaging Neuroscience, Institute of Neurology, London, UK). Standardized uptake value ratio (SUVR) images were created using inferior cerebellar gray as reference region.[2] SUVR images were smoothed to reach a final resolution of 8mm^3^ and warped to Montreal Neuroimaging Institute (MNI) template. Smoothed and warped SUVR images were then converted to W-score maps that quantify the difference between patient 1’s scan and a group of 88 amyloid-PET-negative cognitively unimpaired controls (age=66.14$\pm$19.86).[3] W-scores are age-adjusted Z-score (mean=0, SD=1 in the control group in each voxel).

**Neuropathologic Assessment**

Brain autopsies were performed using 72-hour fixation of freshly cut 1 cm-thick coronal slabs (Case 1) or whole brain immersion fixation for 3 weeks (Case 2). Following fixation, regional dissections were carried out using a previously described protocol to enable tissue sampling of 50 discrete brain regions relevant to the differential diagnosis of dementia based on published consensus criteria.[5–7, 9] Blocks were embedded in paraffin wax and cut into eight micron-thick sections. Staining included hematoxylin and eosin (H&E) on all regions, to enable assessment of the regional neurodegeneration landscape, cerebrovascular disease, and vascular brain injury (including lacunes and microinfarcts), following consensus approaches.[6] Immunohistochemistry for Aβ, hyperphosphorylated tau, α-synuclein, ubiquitin, and transactive response DNA binding protein 43 (TDP-43) was carried out to identify the diagnostic lesions observed in the most common neurodegenerative diseases. Alzheimer’s disease-related changes were assessed according to the Thal amyloid phase, Braak neurofibrillary tangle (NFT) stage, and Consortium to Establish a Registry for Alzheimer’s Disease (CERAD) plaque score. Overall severity of AD neuropathological change (ADNC) was assigned using the National Institute on Aging (NIA) – Reagan criteria and NIA-Alzheimer Association criteria for AD. Coexisting cerebral amyloid angiopathy (CAA), vascular brain injury (VBI), arteriosclerosis, atherosclerosis, argyrophilic grain disease (AGD), Lewy body disease (LBD), and limbic age-related TDP-43 encephalopathy (LATE)[6] were noted when present in the available materials.

**Supplemental Results**

**Neuropathology Results**

**Table 1** Neuropathological Staging

| **Patient** | **ADNC[6]** | **Thal Amyloid Plaque Phase** | **Braak Neurofibrillary Tangle Stage** | **CERAD Neuritic Plaque Score** | **Cerebral Amyloid Angiopathy[9]** | **Lewy Body Disease[5]** | **PD Braak Stage** | **LATE- NC[7]** | **Vascular Brain Injury** |
| --- | --- | --- | --- | --- | --- | --- | --- | --- | --- |
| 1 | Intermediate (A2, B3, C3) | Phase 3 | Stage 5 | Frequent | Mild (CA1/subiculum)** | None | Stage 0 | None | Chronic microinfarcts with gliosis* |
| 2 | High (A3, B3, C3) | Phase 5 | Stage 6 | Frequent | Moderate (frontal, temporal, parietal, cerebellum)** | Diffuse neocortical | Stage 6 | Stage 1 | absent |

* involving subcortical white matter and deep gray nuclei

** capillary CAA not present

| **REGIONS EXAMINED** |
| --- |
| Frontal pole |
| Anterior orbital gyrus |
| Anterior cingulate cortex |
| Middle frontal gyrus |
| Inferior frontal gyrus, pars opercularis |
| Subgenual cingulate cortex |
| Precentral gyrus |
| Superior frontal sulcus |
| Middle insula |
| Entorhinal cortex |
| Inferior temporal gyrus |
| Superior/middle temporal gyrus |
| Postcentral gyrus |
| Posterior cingulate cortex |
| Angular Gyrus |
| Striate cortex |
| Amygdala |
| Dentate gyrus |
| CA3-4 |
| CA2 |
| CA1/Subiculum |
| Ventral striatum |
| Putamen |
| Globus Pallidus |
| Subthalamic nucleus |
| Thalamus |
| Claustrum |
| Dentate nucleus |
| Folia |
| Substantia Nigra |
| Tectum |
| Periaqueductal gray |
| Dorsal raphe |
| Oculomotor nucleus |
| Locus ceruleus |
| Median raphe |
| Abducens nucleus |
| Facial nucleus |
| Nucleus raphe interpositus |
| Hypoglossal nucleus |
| Dorsal efferent nucleus, vagus |
| Nucleus solitary tract |
| Olive |
| Cervical anterior horn cells |
| Cervical corticospinal tract |
| Thoracic anterior horn cells |
| Thoracic intermediolateral cell column |
| Lumbar anterior horn cells |
| Sacral nucleus of Onuf |
| Sacral anterior horn cells |

**Supplemental References**

1. Klunk WE, Koeppe RA, Price JC, Benzinger TL, Devous MD, Jagust WJ, Johnson KA, Mathis CA, Minhas D, Pontecorvo MJ, Rowe CC, Skovronsky DM, Mintun MA (2015) The Centiloid Project: standardizing quantitative amyloid plaque estimation by PET. Alzheimers Dement 11:1-15.e1–4. doi: 10.1016/j.jalz.2014.07.003

2. La Joie R, Bejanin A, Fagan AM, Ayakta N, Baker SL, Bourakova V, Boxer AL, Cha J, Karydas A, Jerome G, Maass A, Mensing A, Miller ZA, O’Neil JP, Pham J, Rosen HJ, Tsai R, Visani AV, Miller BL, Jagust WJ, Rabinovici GD (2018) Associations between [18F]AV1451 tau PET and CSF measures of tau pathology in a clinical sample. Neurology 90:e282–e290. doi: 10.1212/WNL.0000000000004860

3. La Joie R, Perrotin A, Barré L, Hommet C, Mézenge F, Ibazizene M, Camus V, Abbas A, Landeau B, Guilloteau D, de La Sayette V, Eustache F, Desgranges B, Chételat G (2012) Region-specific hierarchy between atrophy, hypometabolism, and β-amyloid (Aβ) load in Alzheimer’s disease dementia. J Neurosci 32:16265–16273. doi: 10.1523/JNEUROSCI.2170-12.2012

4. Landau SM, Fero A, Baker SL, Koeppe R, Mintun M, Chen K, Reiman EM, Jagust WJ (2015) Measurement of Longitudinal β-Amyloid Change with 18F-Florbetapir PET and Standardized Uptake Value Ratios. J Nucl Med 56:567–574. doi: 10.2967/jnumed.114.148981

5. McKeith IG (2006) Consensus guidelines for the clinical and pathologic diagnosis of dementia with Lewy bodies (DLB): report of the Consortium on DLB International Workshop. J Alzheimers Dis 9:417–423. doi: 10.3233/jad-2006-9s347

6. Montine TJ, Phelps CH, Beach TG, Bigio EH, Cairns NJ, Dickson DW, Duyckaerts C, Frosch MP, Masliah E, Mirra SS, Nelson PT, Schneider JA, Thal DR, Trojanowski JQ, Vinters HV, Hyman BT (2012) National Institute on Aging-Alzheimer’s Association guidelines for the neuropathologic assessment of Alzheimer’s disease: a practical approach. Acta Neuropathol 123:1–11. doi: 10.1007/s00401-011-0910-3

7. Nelson PT, Dickson DW, Trojanowski JQ, Jack CR, Boyle PA, Arfanakis K, Rademakers R, Alafuzoff I, Attems J, Brayne C, Coyle-Gilchrist ITS, Chui HC, Fardo DW, Flanagan ME, Halliday G, Hokkanen SRK, Hunter S, Jicha GA, Katsumata Y, Kawas CH, Keene CD, Kovacs GG, Kukull WA, Levey AI, Makkinejad N, Montine TJ, Murayama S, Murray ME, Nag S, Rissman RA, Seeley WW, Sperling RA, White CL, Yu L, Schneider JA (2019) Limbic-predominant age-related TDP-43 encephalopathy (LATE): consensus working group report. Brain 142:1503–1527. doi: 10.1093/brain/awz099

8. Royse SK, Minhas DS, Lopresti BJ, Murphy A, Ward T, Koeppe RA, Bullich S, DeSanti S, Jagust WJ, Landau SM, for the Alzheimer’s Disease Neuroimaging Initiative (2021) Validation of amyloid PET positivity thresholds in centiloids: a multisite PET study approach. Alzheimer’s Research & Therapy 13:99. doi: 10.1186/s13195-021-00836-1

9. Vonsattel JP, Myers RH, Hedley-Whyte ET, Ropper AH, Bird ED, Richardson EP (1991) Cerebral amyloid angiopathy without and with cerebral hemorrhages: a comparative histological study. Ann Neurol 30:637–649. doi: 10.1002/ana.410300503
